# Радиотераностика в эндокринологии и онкоэндокринологии

**DOI:** 10.14341/probl12731

**Published:** 2021-02-04

**Authors:** П. О. Румянцев

**Affiliations:** Национальный медицинский исследовательский центр эндокринологии

**Keywords:** радиотераностика, ПЭТ, ОФЭКТ, эндокринология, онкология

## Abstract

Радиотераностика -  радионуклидная терапия на основе результатов молекулярной визуализации различными радиофармпрепаратами, позволяющие in vivo визуализировать (ОФЭКТ, ПЭТ), а затем системно и при этом селективно воздействовать на патологические метаболические процессы, вызванные патологическим процессом. В последние годы, благодаря успехам в развитии ядерной медицины (рост числа циклотронов, ОФЭКТ/КТ и ПЭТ/КТ в мед. учреждениях) и, прежде всего, радиофармацевтики - в мире радиотераностика развивается очень бурно. Появление новых радиолигандов на основе 177Lu, 131I, 225Ac и других радиоизотопов в мире проводится огромное число клинических исследований радиолигандной терапии нейроэндокринных и хромаффинных опухолей, рака простаты и др. Радиотераностика как новое и очень перспективное направление ядерной медицины прекрасно интегрируется в современные алгоритмы диагностики в области эндокринологии и онкологии. Методы интраоперационной радионавигации позволяют повысить эффективность и безопасность хирургических методов, дистанционной лучевой терапии, брахитерапии. На мой взгляд, будущее персонализированной медицины во многом определит интеграция радиотераностики, мультимодальной визуализации, интраоперационной навигации и существующих/новых методов диагностики и лечения, в сочетании с прикладными геномными и постгеномными технологиями.

## Abstract (en)

Radiotheranostics is a radionuclide therapy based on the results of molecular imaging with various radiopharmaceuticals, allowing in vivo whole body imaging (SPECT, PET), and then systemically and at the same time selectively acting on pathological metabolic processes caused by a pathological process. In recent years, thanks to advances in the development of nuclear medicine (an increase in the number of cyclotrons, SPECT / CT and PET / CT in medical institutions) and, particularly, novel radiopharmaceuticals — in the world, radiotheranostics is developing very rapidly. The emergence of new radioligands labelled by 177Lu, 131I, 225Ac and other radioisotopes in the world have initiated a large number of clinical trials of radioligand therapy of neuroendocrine and chromaffin tumors, prostate cancer, etc. Radiotherapy as a new and very promising direction of nuclear medicine and it is perfectly integrated into modern diagnostic algorithms in the field of endocrinology and oncology. Methods of intraoperative radionavigation make it possible to increase the efficiency and safety of surgical methods, external beam radiation therapy, and brachytherapy. In my opinion, the future of personalized medicine will be predominantly determined by the integration of radiotherapy, multimodal imaging, intraoperative navigation and existing/new methods of diagnosis and treatment, in combination with applied genomic and post-genomic technologies.

Радиоизотопная (молекулярная) визуализация патологии щитовидной железы в 1940-х гг. положила начало и определила вектор развития ядерной медицины во всем мире. Именно эндокринологи первыми применили радиоактивный йод для лечения тиреотоксикоза и рака щитовидной железы, положив начало радиотераностике как таковой. Под термином «тераностика» имеется в виду терапия, основанная на диагностике. С этой точки зрения, наверное, любую терапию можно считать тераностикой, потому как ни одна терапия, по сути, не обходится без диагностики. Принципиальный нюанс радиотераностики в том, что радиоизотопная визуализация позволяет с помощью методов однофотонной эмиссионной томографии (ОФЭКТ) и/или позитронной эмиссионной томографии (ПЭТ) всего тела обнаружить in vivo очаг(и) болезни, с помощью гибридных методов ПЭТ/КТ, ПЭТ/МРТ, ОФЭКТ/КТ установить их анатомическую локализацию и особенности метаболизма, а с применением методов объектной (воксельной) дозиметрии оценить динамику накопления/выведения диагностического радиофармпрепарата (РФП), что в итоге позволит провести системную радионуклидную терапию с расчетом на то, что очаги опухоли селективно накопят и удержат терапевтический РФП, точно так же, как они это сделали с диагностическим (табл. 1)

**Table:** Таблица 1. Радиотераностика в эндокринологии и онкоэндокринологии

Клетки опухоли	Метаболический механизм	РФП
РФП диагностический	РФП терапевтический
Фолликулярные клетки ЩЖ (А- и В-клетки)	Захват йода	ОФЭКТ: 99Tcm-пертехнетат, 123I ПЭТ: 124I	131I
Парафолликулярные клетки ЩЖ (С-клетки)	Метаболизм дофамина, рецепторы соматостатина	ОФЭКТ: 99Tcm-тектротид ПЭТ: 18F-DOPA	-
Паращитовидные железы	Высокая активность митохондрий	ОФЭКТ: 99Tcm-MIBI (технетрил) ПЭТ: 18F-холин	-
Мозговое вещество надпочечников, параганглиома (хромаффинные опухоли)	Синтез норадреналина	ОФЭКТ: 123I-МЙБГ* ПЭТ: 124I-МЙБГ	131I -МЙБГ
Кора надпочечников (кортикостерома, альдостерома, ВДКН)	Метаболизм холестерина, хемокиновые рецепторы	ОФЭКТ: 131I-6-йодметил-19-норхолестерол ПЭТ: 11C-метомидат, 68Ga-пентиксафор	177Lu-Пентиксатер
Гипофиз	Рецепторы к соматостатину	ОФЭКТ: 99Tcm-тектротид ПЭТ: 68Ga-DOTA-TATE (-TOC, -NOC)	177Lu-DOTA-TATE (-TOC)
Нейроэндокринные опухоли (ЖКТ, легкие и другие локализации)	Рецепторы к соматостатину
Инсулиномы, гиперинсулинизм	ГПП-1, GLP-1	ОФЭКТ: 99Tcm-exendin-4 ПЭТ: 68Ga-exendin-4	-
Костный метаболизм	Захват кальция	ОФЭКТ: 99Tcm-технефор и аналоги ПЭТ: 18F-флюорид натрия	223RaСl2 153Sa-оксабифор 89Sr Сl2
Потенциал злокачественности	Метаболизм глюкозы	ПЭТ: 18F-фтордезоксиглюкоза	-
Опухоли эстроген-секретирующие	Экспрессия рецепторов эстрогенов	ПЭТ: 18F-флюоро-эстрадиол	-

ВДКН — врожденная дисплазия коры надпочечников; ГПП-1 — глюкагоноподобный пептид 1 типа; ЖКТ — желудочно-кишечный тракт; МЙБГ — метайодбензилгуанидин; РФП — радиофармпрепарат; ЩЖ — щитовидная железа.

В течение последних семи десятилетий многократно увеличился перечень диагностических и терапевтических радиоизотопов и меченных ими «умных» лигандов (метаболиты, антигены, антитела) для анатомо-метаболической визуализации и последующей прецизионной радионуклидной терапии не только эндокринных и онкоэндокринных заболеваний, но и других болезней (онкология, кардиология, неврология).

## МОЛЕКУЛЯРНАЯ ВИЗУАЛИЗАЦИЯ РАЗЛИЧНЫХ НАРУШЕНИЙ МЕТАБОЛИЗМА

ПЭТ позволяет in vivo изучать различные нарушения метаболизма с помощью целенаправленных химических соединений, меченных позитрон-излучающими радиоизотопами. Разработка метода исследования заключается в выборе молекулы интереса (субстрат или аналог) и эффективного способа ее мечения атомами 11C, 18F или др. Молекулярный субстрат, меченный радиоизотопом, подвергается в организме фермент-опосредованной метаболической трансформации с последующим накоплением в тканях, что можно оценить во времени и количественно (стандартизованный уровень накопления, SUV) c помощью ПЭТ. Так как захват клетками таргетного молекулярного субстрата опосредуется специфическими ферментами (такими, как хемокиназы, тимидин-киназы и т.п.), накопление радиометки в тканях отражает степень ферментативной экспрессии в ней.

## ПЭТ-ВИЗУАЛИЗАЦИЯ МЕТАБОЛИЗМА ГЛЮКОЗЫ

Наиболее важной молекулой для обеспечения клеток энергией для множества биохимических реакций является аденозинтрифосфат (АТФ), который вырабатывается в митохондриях вследствие метаболизма глюкозы путем окислительного фосфорилирования в целях энергетического обеспечения клеточных процессов жизнедеятельности. Клетки головного мозга работают исключительно на энергии расщепления АТФ, в то время как метаболизм миокарда и жировой ткани является главным источником выработки АТФ. Повышенный метаболизм глюкозы обнаружен в клетках опухолей, и он основан на аэробном гликолизе, или феномене так называемого «эффекта Варбурга», при котором интенсивный метаболизм глюкозы сохраняется даже в условиях гипоксии. Глюкоза в основном транспортируется в клетку путем диффузии через специфические транспортеры в клеточной мембране, а в опухолевых клетках — преимущественно через транспортер GLUT-1. В цитозоле глюкоза конвертируется путем фосфорилирования с участием фермента хемокиназы в глюкозо-6-фосфат, который в последующем метаболизируется в диоксид углерода и воду. Было показано, что гидроксильная группа на втором атоме углерода не обязательна для фосфорилирования хемокиназой. Дезоксиглюкоза проникает в клетку и конвертируется в дезоксиглюкозо-6-фосфат, однако не подвергается дальнейшему катаболизму, накапливаясь в клетке. Для ПЭТ/КТ в качестве РФП используется молекула 2-дезокси-2-фтор-дезоксиглюкозы (ФДГ), в которой атом водорода во второй позиции заменен на 18F для молекулярной визуализации метаболизма глюкозы. При этом связь атомов углерода и фтора даже более стабильна, чем связь углерода и водорода, и химические нераспознаваема хемокиназой. В результате в клетках, помимо глюкозы, накапливается введенный в качестве РФП ФДГ-6-фосфат. В опухолевых клетках повышенный гликолиз и высокий захват ФДГ также связаны с усилением работы вышеупомянутого транспортера глюкозы GLUT-1 (инсулинзависимого) и внутриклеточной экспрессии хемокиназы.

## ПЭТ-ВИЗУАЛИЗАЦИЯ МЕТАБОЛИЗМА ЖИРНЫХ КИСЛОТ

Продукция АТФ миокардом базируется на современных знаниях о метаболизме молекулярных субстратов (свободных жирных кислот, глюкозы, лактата, кетоновых телец), а также биодоступности кислорода и уровне различных гормонов (катехоламинов, инсулина, глюкагона и др.). Когда уровень глюкозы в плазме возрастает, миокард переключается на метаболизм глюкозы, и, как результат, 18F-ФДГ может использоваться для оценки утилизации миокардом глюкозы. Натощак, когда уровень инсулина в крови низкий, уровень свободных жирных кислот повышается, а доля вырабатываемой клетками АТФ на основе окисления свободных жирных кислот достигает 70–80%. Фермент тиокиназа в цитозоле конвертирует свободные жирные кислоты в ацетил-коэнзим-А, который впоследствии подвергается β-оксигенации в митохондриях и расщепляется на двухуглеродные фрагменты, метаболизирующиеся в цикле трикарбоновых кислот. Меченная 11C 16-углеродная цепочка пальмитата (1-[11C]-palmitate) в цитозоле клеток конвертируется в 11C-ацетил-коэнзим-А. Часть из них попадает в митохондриях в цикл трикарбоновых кислот для окисления, в то время как остаточная порция инкорпорируется в триглицериды и фосфолипидный пул в цитозоле. Так как метаболизм пальмитата сложный, был синтезирован его метаболический аналог свободных жирных кислот (тиагептадекановая кислота), меченный 18F. РФП известен как 14(R,S)-[18F]-6-тиагептадекановая кислота (FTHA), разработанный для визуализации метаболического захвата миокардом с минимальной фоновой диффузией РФП в кровь.

## ПЭТ-ВИЗУАЛИЗАЦИЯ МЕТАБОЛИЗМА ХОЛИНА

Все клетки утилизируют холин в качестве предшественника для биосинтеза фосфолипидов, который является важнейшим компонентом клеточных мембран. Внутри клетки холин может фосфорилироваться, окисляться или подвергаться ацетилированию. Фосфорилирование холина катализируется ферментом холинкиназой. Фосфохолин является внутриклеточным депо холина и в дальнейшем используется для синтеза лецитина (фосфатидилхолина), главного фосфолипида всех клеточных мембран. Гормональная гиперфункция и канцерогенез характеризуются повышением клеточной пролиферации. Было установлено, что эти процессы связаны с индукцией активности холинкиназы, приводящей к увеличению внутриклеточного фосфохолина. Более того, хорошо известно, что быстро пролиферирующие гормонально-активные и опухолевые клетки содержат огромное количество фосфолипидов, особенно фосфатидилхолина. Сегодня РФП 11C-холин применяется для ПЭТ-диагностики заболеваний головного мозга и рака предстательной железы. Одной из главных проблем 11C-холина является его метаболизм в крови, где он конвертируется в 11C-бетаин, снижающий чувствительность и специфичность диагностики. Для снижения метаболической трансформации РФП в крови были предложены различные, меченные 18F, метаболические аналоги холина, такие как 18F-фтор-метилхолин и 18F-фтор-этилхолин, обеспечивающие высокий захват в патологических опухолевых гиперфункционирующих очагах, костной ткани, метастазах, что расширило спектр его клинического применения, особенно в онкоэндокринологии.

## ПЭТ-ВИЗУАЛИЗАЦИЯ КОСТНОГО МЕТАБОЛИЗМА

Благодаря своей высокой аффинности к кальцию, 18F-флуорид натрия является идеальным для оценки костного метаболизма. 18F-флуорид натрия продемонстрировал высокую способность захвата костной ткани при хорошем клиренсе в кровотоке, что ведет к повышению контрастности ПЭТ-визуализации костной ткани (повышение соотношения кость-фон). ПЭТ с 18F-флуоридом натрия широко используется для оценки костного метаболизма в онкоэндокринологии при раке щитовидной железы, опухолях паращитовидных железы, остеолитическом синдроме вследствие неоплазии фактора роста фибробластов FR23, орфанных эндокринопатиях и других эндокринных заболеваниях, осложненных остеопатиями. Данный метод успешно дополняет остеосцинтиграфию (ОФЭКТ), иллюстрируя активность остеобластов, позволяя осуществлять молекулярную визуализацию остеолитических процессов в костной ткани.

## МЕТАБОЛИЧЕСКАЯ ВИЗУАЛИЗАЦИЯ МИОКАРДА

Метаболическая визуализация перфузии кислорода, обмена калия и различных органических метаболитов (глютамин и пр.) в клетках миокарда с помощью ПЭТ позволяет с высокой точностью и разрешающей способностью обнаружить очаги ишемии или иных нарушений в клетках миокарда. В свою очередь, это позволяет распознать на ранней стадии и охарактеризовать кардиальную патологию, обеспечить контролируемую с помощью ПЭТ профилактику и лечение различных кардиальных патологий, риск развития которых значительно повышен при эндокринных заболеваниях (сахарный диабет, тиреотоксикоз, гиперкортицизм и пр.).

Кардиологические ПЭТ-исследования выполняются в трехмерном (3D) формате, очень иллюстративны, используются в качестве метода экспертной и предельно точной диагностики нарушений перфузии и метаболических нарушений миокарда. Исследования проводятся в покое, под физической нагрузкой (тредмил), а также с лекарственными пробами. Лучевая нагрузка при ПЭТ небольшая и составляет 1,7–8 мЗв.

Радиоактивные изотопы и РФП на их основе, используемые для ПЭТ-кардиологии, имеют различный период полураспада (от 2 до 109 мин). Радиоактивный изотоп рубидия-82 (82Rb) является биологическим аналогом калия. Широко используется в неотложной и плановой ядерной кардиологии (ПЭТ/КТ) в США и Европе для визуализации острых и скрытых нарушений перфузии миокарда. В биологической основе метода лежит оценка функционирования натрий-калиевой АТФазы. При исследовании оценивается активность работы натрий-калиевого насоса с помощью РФП и миокардиальной перфузии, в частности, на основе азота-13 (13N-ammonia), который накапливается в кардиомиоцитах для ферментативной конверсии в глютамин с помощью глутамин-синтетазы. Данный РФП может также применяться для молекулярной визуализации опухолей головного мозга. Для динамических перфузионных ПЭТ-исследований также применяется 15-кислород (15O) в форме монооксида и диоксида. Еще одним РФП для ПЭТ-кардиологии является фторпиридаз на основе 18F (18F-flurpiridaz). Мишенью для данного РФП являются митохондрии кардиомиоцитов, активность (захват клетками РФП) отражает их функциональную активность. В сочетании с методом МРТ (ПЭТ/МРТ) преумножается пространственное разрешение и снижается частота артефактов аттенуации. Закономерно возрастает точность и повышается качество стратификации риска. Использование ПЭТ/КТ позволяет оценить индекс кальцинации сосудов.

## МОЛЕКУЛЯРНАЯ ВИЗУАЛИЗАЦИЯ МЕТАБОЛИЗМА ГОЛОВНОГО МОЗГА

Мультимодальная визуализация с помощью гибридизации методов МРТ и ПЭТ позволяет на молекулярно-метаболическом уровне обнаружить патологические процессы в головном мозге, а также оценить их динамику на фоне лечения (табл. 2).

**Table:** Таблица 2. Диагностические возможности методов магнитно-резонансной и позитронно-эмиссионной томографии и ПЭТ

МРТ	ПЭТ
Морфология (режимы Т1, Т2, PD, IR)	Скорость кровотока (H215O)
Диффузно-взвешенная визуализация (DWI, DTI, ADC)	Метаболизм глюкозы (18F-FDG)
Перфузионно-взвешенная визуализация (ASL, DSC, DCE)	Отложение белка (напр., 11С-PIB)
Функциональная визуализация (BOLD)	Аминокислоты (11С-метионин/18FET)
Сосудистая анатомия (МР-ангиография)	Ферментативная активность (например, 11С-MP4A)
Метаболическая МР-спектроскопия	Нейротрансмиссия (например, 11С-raclopride)

С помощью ПЭТ/КТ и ПЭТ/МРТ становится возможной in vivo визуализация метаболических путей допамина (DOPA), в том числе допаминовых транспортеров, бета-амилоида, D-рецепторов (D1, D2, D3), аспартата (NMDA, N-метил-D-аспартата), P-гликопротеина, холинергической системы, глютамат-5-рецепторов, различных нейротрансмиттеров (VMAT2, DTBZ и др.), аденозиновых рецепторов, серотониновой и норадренергических систем (рецепторы, транспортеры), моноаминоксидазной системы и многие другие.

Сегодня многими ПЭТ воспринимается как более современная технология радиоизотопной диагностики, чем ОФЭКТ. Некоторые даже считают, что ПЭТ по этой причине со временем вытеснит ОФЭКТ из клинической практики. Это в корне ошибочное представление. Метод ПЭТ действительно обладает более высокой чувствительностью и разрешающей способностью визуализации, однако по специфичности методы значимо не различаются. При этом ПЭТ, например, не может быть использована для дозиметрического планирования и оценки эффективности радионуклидной терапии, это «юрисдикция» ОФЭКТ. Не уходя в глубины сравнительного анализа, хочется прояснить, что это комплементарные методы молекулярной визуализации, прекрасно дополняющие и развивающие друг друга, особенно в концепции радиотераностики.

Радиотераностика — радионуклидная терапия на основе результатов молекулярной визуализации с помощью различных РФП, позволяющая визуализировать in vivo (ОФЭКТ, ПЭТ) и селективно воздействовать на патологические метаболические процессы, вызванные опухолью. Используя эту парадигму, с 1950-х гг. прошлого столетия с помощью радиоактивного йода успешно лечатся тиреотоксикоз и рак щитовидной железы. В последние годы, благодаря успехам в развитии ядерной медицины (рост числа циклотронов, ОФЭКТ/КТ и ПЭТ/КТ в медицинских учреждениях) и, прежде всего, радиофармацевтики, в мире очень бурно развивается радиотераностика. Появление новых радиолигандов на основе 177Lu, 225Ac и других радиоизотопов стимулировало большое количестве (более 300) клинических исследований по радиолигандной терапии рака простаты, нейроэндокринных опухолей, рака поджелудочной железы и других злокачественных новообразований. Одним из самых перспективных направлений радиотераностики является разработка радиолигандов на основе таргетных противоопухолевых препаратов, что позволяет суммировать в одном РФП два эффекта: ингибирование сигнальных каскадов и лучевое повреждение.

Радиотераностика может служить как самостоятельным (например, тиреотоксикоз), так и вспомогательным, адъювантным (например, рак щитовидной железы, нейроэндокринные опухоли, гормонально-активные опухоли и пр.) методом лечения различных эндокринных и онкологических заболеваний. Важнейшими ее преимуществами являются системный и при этом избирательный терапевтический эффект, возможность сочетания с другими методами лечения — фокальными (хирургия, дистанционная лучевая терапия, брахитерапия, термоаблация и пр.) и системными (химиотерапия, таргетная и иммунотерапия) — с приоритетом повышения эффективности таковой комбинации и без повышения токсичности.

Методы фокальной радионуклидной визуализации сегодня активно внедряются в хирургическую практику, позволяя по принципу миноискателя «подсветить» очаги опухоли, оценить индивидуальные особенности лимфооттока (сторожевые лимфатические узлы). Интраоперационная радионавигация, на мой взгляд, в сочетании с предоперационной молекулярной визуализацией развивает хирургию в сторону более высокой эффективности и безопасности.

Самой отличительной особенностью молекулярной визуализации и радиотераностики является возможность увидеть in vivo патологический процесс и оценить его метаболизм, потенциальную угрозу для здоровья и жизни пациента, продуманно подбирать тактику ведения, оперативно корректировать в процессе лечения, оценивая ее эффективность. При этом прогноз эффективности и потенциальной токсичности можно оценить уже на диагностическом этапе (ОФЭКТ/КТ, ПЭТ/КТ), а управление данными параметрами невозможно без разработки и внедрения индивидуальных дозиметрических и генетических (радиогеномных) и метаболомных (биомаркеры) исследований. Так, установление наследственной предрасположенности к развитию заболевания позволяет прогнозировать риск ее развития в течение жизни, а биомаркеры и методы визуализации — обнаружить болезнь на ранней стадии и установить ее локализацию/стадию, своевременно выполнить адекватное лечение.

